# Automated fluence optimization in breast cancer radiotherapy: balancing dosimetric quality, organ-at-risk sparing, and workflow efficiency

**DOI:** 10.1186/s13014-026-02815-y

**Published:** 2026-03-04

**Authors:** Young Eun Choi, Han-Back Shin, KiHoon Sung

**Affiliations:** 1 Department of Radiation Oncology, Gachon University Gil Medical Center, Incheon, Republic of Korea; 2 Department of Radiation Oncology, Gachon University College of Medicine, Incheon, Republic of Korea

**Keywords:** Automated treatment planning, Breast cancer radiotherapy, Contralateral breast exposure, Cardiac sparing, EZFluence, Fluence optimization

## Abstract

**Background:**

In this study, we evaluated the effectiveness of EZFluence (EZF), an automated fluence optimization tool, in planning breast cancer radiotherapy by comparing its dosimetric performance with that of three-dimensional conformal radiotherapy (3D-CRT), intensity-modulated radiation therapy (IMRT), and volumetric-modulated arc therapy (VMAT). Specifically, we aimed to determine whether EZF can enhance target coverage, minimize dose to organs at risk, and improve planning efficiency.

**Methods:**

This retrospective analysis included 35 patients who received whole-breast irradiation for left-sided breast cancer. Treatment plans were generated using 3D-CRT, IMRT, VMAT, and EZF-based forward-planned field-in-field techniques. Dosimetric parameters, including planning target volume coverage, cardiac exposure, left anterior descending artery dose, lung dose, and contralateral tissue dose, were analyzed. Planning efficiency was assessed by comparing the monitor units and planning time.

**Results:**

EZF demonstrated significantly superior dose homogeneity and a lower delivered maximum dose compared with 3D-CRT and IMRT. Although VMAT provided the highest planning target volume V_95_, EZF maintained comparable target coverage with significantly reduced high-dose regions. Additionally, EZF achieved a lower mean heart dose compared with 3D-CRT and IMRT. While VMAT offered superior high-dose cardiac sparing, EZF minimized low-dose cardiac spread and significantly reduced the mean lung dose and low-dose volumes of the left lung compared with IMRT and VMAT. The mean doses to the contralateral breast with 3D-CRT and EZF were less than 1 Gy, whereas those when using IMRT and VMAT exceeded 1 Gy. Regarding efficiency, EZF required fewer monitor units than did IMRT and VMAT, and its automated planning process reduced inter-planner variability.

**Conclusion:**

EZF demonstrated significant potential as an innovative approach in breast cancer radiotherapy, providing comparable target coverage with enhanced sparing of organs at risk and without unnecessary contralateral dose. Its automated fluence optimization enhanced planning efficiency by simplifying planning and reducing variability. These findings support the integration of EZF into routine clinical practice to optimize radiotherapy delivery.

**Supplementary Information:**

The online version contains supplementary material available at 10.1186/s13014-026-02815-y.

## Background

Breast cancer is a leading cause of cancer-related morbidity and mortality among women worldwide [1]. However, earlier detection and advances in treatment modalities in recent years have contributed to significant improvements in patient survival. Treatment for early-stage breast cancer typically involves a combination of surgery, radiation therapy, and systemic therapies, among which radiotherapy makes an essential contribution to eradicating residual microscopic disease and reducing the risk of local recurrence [2, 3].

Three-dimensional conformal radiotherapy (3D-CRT) has been used for whole-breast irradiation for decades as a standard conventional technique, providing simple and effective coverage of the target volume. However, it often results in suboptimal dose conformity and increased exposure of organs at risk (OARs), such as the heart and ipsilateral lung, thus leading to concerns regarding late toxicities [2, 4, 5]. To address these limitations, intensity-modulated radiation therapy (IMRT) and volumetric-modulated arc therapy (VMAT) have been introduced as advanced techniques with the aim of enhancing dose conformity, reducing dose heterogeneity, and sparing unaffected tissues [6–9].

However, despite their dosimetric advantages, IMRT and VMAT are not without certain drawbacks. Both require more complex planning and optimization, thereby leading to increased planning time and inter-planner variability. Although these techniques effectively reduce high-dose exposure to the heart and lungs, their use is often associated with an increase in the spread of low-dose radiation, which may contribute to the risk of secondary malignancy, particularly in the contralateral breast [8, 10]. Automated treatment planning systems have recently emerged as promising solutions to these limitations. These technologies encompass a broad spectrum of innovations, ranging from script-based and knowledge-based planning strategies that standardize plan quality [11, 12], to fully automated VMAT techniques designed for complex cases such as locally advanced breast cancer [13]. Furthermore, recent advancements in deep learning-driven algorithms continue to enhance dose prediction and optimization efficiency [14]. By reducing the dependence on manual optimization of treatment plans, these systems enhance workflow efficiency, minimize variability between planners, and improve treatment consistency [15]. Forward-planned field-in-field treatment planning software, specifically, EZFluence (EZF; version 2.4.1; Radformation Inc., New York, NY, USA), has gained considerable attention because of its ability to automate fluence optimization and maintain high dosimetric quality. Compared with the more traditional planning methods, EZF enables planners to achieve better dose homogeneity, reduce planning time, and optimize OAR sparing [16–19]. Unlike IMRT and VMAT, which require intricate optimization algorithms, EZF simplifies the treatment planning process and maintains competitive dosimetric outcomes.

Therefore, in this study, we aimed to evaluate the clinical performance of EZF in breast cancer radiotherapy by comparing its effectiveness with that of standard techniques, namely, 3D-CRT, IMRT, and VMAT. By investigating whether EZF can provide comparable or superior treatment quality, as well as simplifying the planning process, the findings of this study can contribute to ongoing advances in radiotherapy and establish the feasibility of integrating automated planning solutions in clinical practice.

## Materials and methods

### Target delineation and anatomical structures

In this study, we retrospectively analyzed 35 patients with left-sided breast cancer who underwent radiotherapy at our institution between January and December 2023. We collected only data from patients with left-sided breast cancer to evaluate the dose of radiation to which the heart was exposed. All patients underwent computed tomography simulation following institutional guidelines, with images being acquired in the free-breathing state at 3-mm slice intervals to ensure high-resolution anatomical visualization. Target delineation and treatment planning were performed using the Eclipse Treatment Planning System (version 17.0.0; Varian Medical Systems, Palo Alto, CA, USA).

The tumor bed was identified based on the presence of surgical clips or an associated seroma, which served as a key reference for defining the clinical target volume (CTV). Thereafter, the planning target volume (PTV) was delineated to encompass the visible breast tissue and tumor bed, whilst taking into consideration anatomical constraints to minimize unnecessary exposure of the surrounding normal tissues to radiation. PTV expansion was restricted to the outer contour of the ribs and extended 5 mm from the skin surface to ensure appropriate dose distribution.

For the delineation of OARs, excluding the left anterior descending artery (LAD), an automated contouring tool (OncoStudio; OncoSoft Inc., Seoul, Republic of Korea) was used to enhance efficiency and consistency. This artificial intelligence based segmentation software facilitates accurate identification of important structures and minimizes inter-observer variability. Using the method described by Taylor et al., a 1-cm radius of the LAD was outlined from the root of the aorta to the apex of the heart [20]. To ensure clinical accuracy, all contours generated using the software were reviewed and manually adjusted by an experienced radiation oncologist.

### Treatment planning

Treatment planning was conducted using the Eclipse Treatment Planning System in combination with a Varian Clinac iX linear accelerator. Each plan was developed based on predefined planning objectives to ensure optimal dose coverage of the PTV and minimize the exposure of OARs. Furthermore, to ensure effective therapeutic delivery and adherence to established clinical guidelines, for all plans, we used a total prescribed dose of 40.05 Gy delivered in 15 fractions.

The 3D-CRT plans were created using wedged tangential fields with 6-MV half-beams to achieve adequate target coverage and limit the unnecessary irradiation of adjacent tissues. The IMRT plans were designed using the same beam angles as used for the 3D-CRT plans, incorporating two opposed tangential 6-MV beams with up to five additional fields manually introduced at angles of 20–30° between the opposed beams to optimize dose conformity. For VMAT planning, we used two fields, for which the starting and ending angles were identical to those used for the 3D-CRT plans. This technique enabled improved dose modulation and provided greater flexibility with respect to optimizing the dose distribution.

Treatment plans developed using EZF, a forward-planned field-in-field type of treatment planning software, were generated using tangential beam arrangements identical to those used to generate the 3D-CRT plans. Having established the beam configuration, the EZF script was executed within Eclipse, extracting relevant treatment plan parameters, including patient anatomy, beam geometry, and dose prescription, with the automated fluence optimization feature being utilized to achieve a more uniform dose distribution based on a systematic adjustment of the fluence maps to minimize hot and cold spots. This system automatically generates subfields based on the initial dose distribution and applies modifications to enhance homogeneity. The final EZF-generated fluence patterns thus obtained were imported into Eclipse Treatment Planning System, which performed the dose calculation. For all techniques, planning objectives and normal tissue dose constraints were established in accordance with the NRG Oncology/RTOG 1005 protocol guidelines [21]. The primary target coverage goal was to ensure that at least 95% of the PTV received 95% of the prescribed dose (V_95%_ ≥ 95%) while limiting the maximum dose D_max_ to < 107% of the prescribed dose. The specific dose constraints for OARs, including the heart, lungs, and contralateral breast, are summarized in Table 1.

**Table 1:** Planning objectives and dose constraints for organs at risk (OARs)

Organs at Risk	Metric	Ideal Criteria	Acceptable Criteria
Heart	V_8Gy_​	< 30%	< 35%
	D_mean_	< 3.2 Gy	< 4.0 Gy
	D_5%​_	< 16 Gy	< 20 Gy
Contralateral breast	D_max_​	< 2.4 Gy	< 2.64 Gy
Ipsilateral lung	V_16Gy_​	< 15%	< 20%
	V_8Gy_​	< 35%	< 40%
	V_4Gy_​	< 50%	< 55%
Contralateral lung	V_4Gy_​	< 10%	< 15%

V_xGy_, volume receiving at least x Gy; D_5%_, dose received by 5% of the volume. Note: These constraints were established in accordance with the NRG Oncology/RTOG 1005 protocol [21]

To evaluate the efficiency of the different planning techniques, the total planning time was recorded and the number of monitor units (MUs) required for treatment delivery was quantified.

To compare the parameters of different planning techniques, we generated dose–volume histograms for each structure. In addition, we calculated values for the following dosimetric parameters: the mean dose (D_mean_), maximum dose (D_max_), relative structure volume receiving greater than or equal to a dose of x Gy (V_xGy_) or x% (V_x_), and the dose received by x% of the structure volume (D_x_). We also evaluated, the dosimetric effects of each of the assessed planning technique on the heart, lungs, ipsilateral lung, contralateral breast, and LAD, and to assess dose homogeneity and target coverage, PTV V_95_, PTV D_max_, and PTV V_105_ were analyzed.

Statistical analyses and data visualization were performed using R statistical software (version 4.4.1; R Foundation for Statistical Computing, Vienna, Austria). A repeated-measures analysis of variance was conducted to compare the dosimetric parameters of the four treatment techniques. If a significant difference was found (*p* < 0.05), post-hoc pairwise comparisons were performed using Bonferroni-corrected *t*-tests to adjust for multiple comparisons. An adjusted p-value < 0.0125 was considered significant after Bonferroni correction.

## Results

### Patient characteristics

The clinical characteristics of the 35 patients included in this study are summarized in Table 2. The median age of the cohort was 61 years (range, 36–79 years). The study population consisted exclusively of patients with early-stage breast cancer. The distribution of tumor stages was as follows: Tis (*n* = 8), T1 (*n* = 23), and T2 (*n* = 4). Crucially, all patients were confirmed to be free of lymph node involvement (N0) and distant metastasis (M0) at the time of diagnosis. Consequently, the clinical target volume for all plans was confined to the whole breast, excluding regional lymph node stations.

**Table 2:** Patients and tumor characteristics

Characteristics	Value (*n* = 35)
Median age (range)	61 years (36–79)
T Stage	
Tis	8 (22.9%)
T1a	3 (8.6%)
T1b	2 (5.7%)
T1c	18 (51.4%)
T2	4 (11.4%)
N Stage	
N0	35 (100%)
M Stage	
M0	35 (100%)
Left breast volume (cm^3^)	
Median (range)	632.9 (253.7-1066.5)
Mean ± SD	657.2 ± 211.9

SD, standard deviation. Note: Values are presented as number (%) unless otherwise indicated

### Target volume coverage and dose homogeneity

**Fig. 1:**
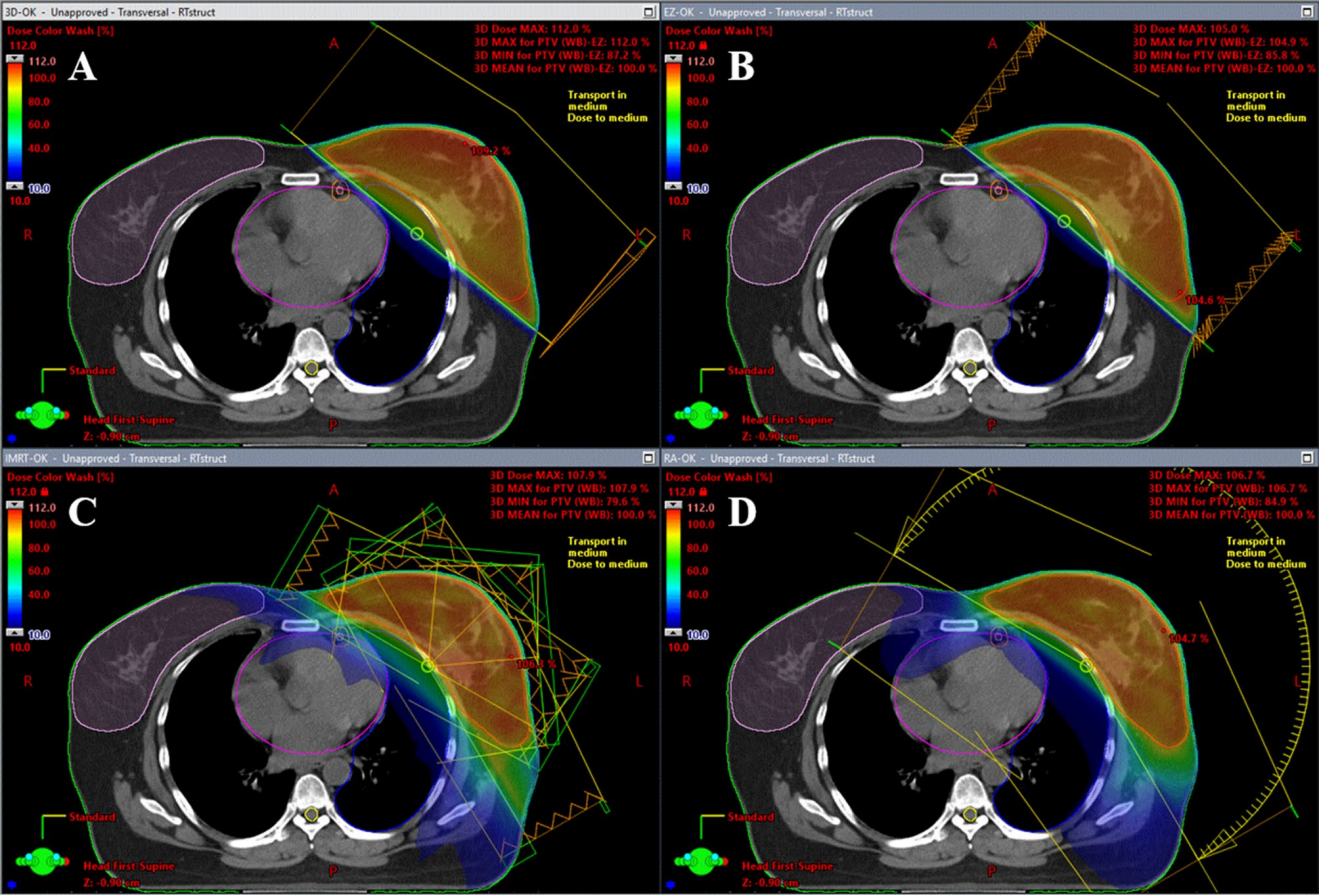
Comparison of dose distributions of breast radiotherapy plans. The figure illustrates the comparison of dose distributions of breast radiotherapy plans for (**A**) 3D-CRT; (**B**) EZF; (**C**) IMRT; and (**D**) VMAT. The color wash overlay represents dose coverage across axial CT slices, thus highlighting variations in target coverage and exposure of organs at risk. 3D-CRT, three-dimensional conformal radiotherapy; EZF, EZFluence; IMRT, intensity-modulated radiation therapy; VMAT, volumetric-modulated arc therapy

**Fig. 2:**
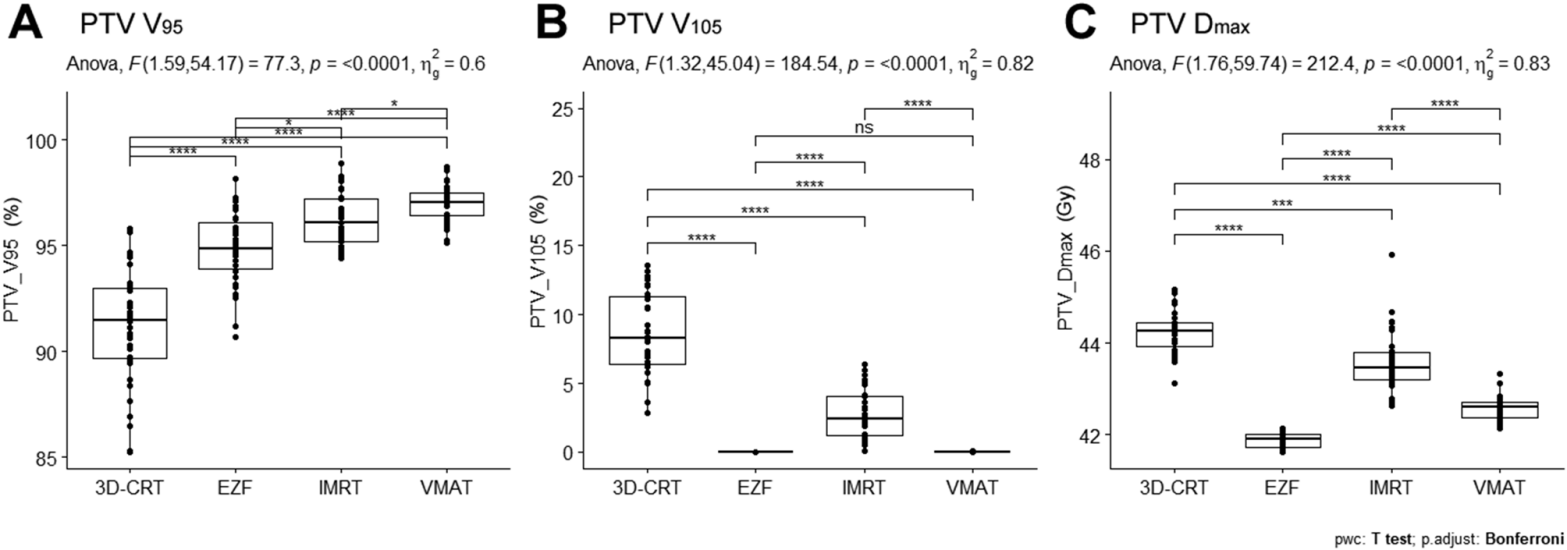
Comparison of dose coverage and dose homogeneity. The figure depicts the comparison of dose coverage and dose homogeneity among different radiotherapy techniques. 3D-CRT, three-dimensional conformal radiotherapy; EZF, EZFluence; IMRT, intensity-modulated radiation therapy; VMAT, volumetric-modulated arc therapy. Significance was considered as follows: *adjusted *p* < 0.05; **adjusted *p* < 0.01; ***adjusted *p* < 0.001; and ****adjusted *p* < 0.0001; ns, not significant

Figure 1 presents a comparative visualization of dose distributions using the four assessed radiotherapy techniques. Detailed comparisons of the average dose-volume histograms (DVHs) for the PTV, CTV, and OARs across the four techniques are illustrated in Supplementary Figure S1. Among these techniques, we detected significant differences with respect to the calculated PTV V_95_ (F = 77.3; *p* < 0.0001), PTV V_105_ (F = 184.54; *p* < 0.0001), and PTV D_max_ (F = 212.4; *p* < 0.0001) values (Table 3; Fig. 2). Specifically, using VMAT, we obtained a PTV V_95_ value that was significantly higher than that obtained using 3D-CRT (adjusted *p* < 0.0001), whereas no significant differences were detected when using IMRT and EZF (adjusted *p* < 0.05). With respect to PTV D_max_, we found that the lowest values were obtained when using EZF, with a statistically significant trend observed in the order EZF < VMAT < IMRT < 3D-CRT (adjusted *p* < 0.001). In addition, compared with IMRT and 3D-CRT, significantly lower PTV V_105_ values were obtained when using VMAT and EZF (adjusted *p* < 0.0001).

**Table 3:** Comparison of dosimetric parameters among different radiotherapy techniques (mean ± standard deviation)

	3D-CRT	EZF	IMRT	VMAT	p*	Adjusted *p*†
PTV						
V_95%_ (%)	91.22 ± 2.80	94.82 ± 1.81	96.17 ± 1.27	97.01 ± 0.88	< 0.0001	a, b,c, e
V_105%_ (%)	8.62 ± 2.88	0	2.69 ± 1.74	0.01 ± 0.02	< 0.0001	a, b,c, d,f
D_max_ (Gy)	44.23 ± 0.46	41.87 ± 0.15	43.58 ± 0.64	42.57 ± 0.25	< 0.0001	a, b,c, d,e, f
Heart						
D_mean_ (Gy)	2.45 ± 0.79	1.63 ± 0.65	4.04 ± 0.61	3.36 ± 0.46	< 0.0001	a, b,c, d,e, f
V_8Gy_ (%)	3.76 ± 2.50	2.80 ± 2.04	9.29 ± 3.58	6.25 ± 2.12	< 0.0001	a, b,c, d,e, f
V_30Gy_ (%)	1.53 ± 1.40	0.99 ± 1.03	0.03 ± 0.08	0.01 ± 0.04	< 0.0001	a, b,c, d,e
D_5%_ (Gy)	8.15 ± 7.42	5.05 ± 4.06	10.70 ± 2.31	8.80 ± 1.39	< 0.001	a, d,e, f
LAD						
D_mean_ (Gy)	10.04 ± 5.09	8.12 ± 4.73	10.40 ± 2.68	8.88 ± 2.23	0.002	a, d,f
D_max_ (Gy)	35.55 ± 9.49	34.87 ± 10.28	33.01 ± 6.37	31.39 ± 6.53	0.003	c, f
Lungs						
D_mean_ (Gy)	2.39 ± 0.71	1.74 ± 0.63	3.48 ± 0.66	3.10 ± 0.61	< 0.0001	a, b,c, d,e, f
V_20Gy_ (%)	3.76 ± 1.73	3.12 ± 1.59	2.52 ± 0.83	1.94 ± 0.65	< 0.0001	a, b,c, e,f
Ipsilateral lung						
V_4Gy_ (%)	20.84 ± 6.75	17.12 ± 5.96	39.07 ± 7.16	34.06 ± 5.20	< 0.0001	a, b,c, d,e, f
V_8Gy_ (%)	13.88 ± 5.33	11.87 ± 4.89	20.70 ± 3.91	15.66 ± 3.06	< 0.0001	a, b,d, e,f
V_16Gy_ (%)	9.82 ± 4.37	8.28 ± 4.01	8.84 ± 2.57	6.61 ± 1.84	< 0.0001	a, c,f
Contralateral breast						
D_mean_ (Gy)	0.53 ± 0.11	0.28 ± 0.07	1.96 ± 0.48	2.25 ± 0.78	< 0.0001	a, b,c, d,e
V_3Gy_ (%)	0	0	18.71 ± 12.87	22.83 ± 14.62	< 0.0001	b, c,d, e
V_10Gy_ (%)	0	0	0.04 ± 0.13	0.00 ± 0.00	0.016	
D_max_ (Gy)	1.96 ± 0.29	1.61 ± 0.30	8.66 ± 2.91	6.75 ± 1.49	< 0.0001	a, b,c, d,e, f
Contralateral lung						
V_4Gy_ (%)	0	0	10.68 ± 8.27	11.71 ± 7.03	< 0.0001	b, c,d, e
MU	436.77 ± 28.38	433.09 ± 40.16	2691.89 ± 387.69	616.17 ± 47.51	< 0.0001	b, c,d, e,f
Planning time (min)	13.98 ± 2.37	7.24 ± 2.06	56.98 ± 9.71	109.72 ± 25.95	< 0.0001	a, b,c, d,e, f

Dose values are presented as Gy. Volume data are shown as percentages. Statistical comparisons of 3D-CRT, EZF, IMRT, and VMAT were performed

* p from the repeated-measure analysis of variance

† Bonferroni-corrected p (adjusted p) < 0.0125: a = 3D-CRT vs. EZF; b = 3D-CRT vs. IMRT; c = 3D-CRT vs. VMAT; d = EZF vs. IMRT; e = EZF vs. VMAT; f = IMRT vs. VMAT

3D-CRT, three-dimensional conformal radiotherapy; EZF, EZFluence; IMRT, intensity-modulated radiation therapy; VMAT, volumetric-modulated arc therapy; PTV, planning target volume; LAD, left anterior descending artery; MU, monitor unit

### Organ at risk sparing

**Fig. 3:**
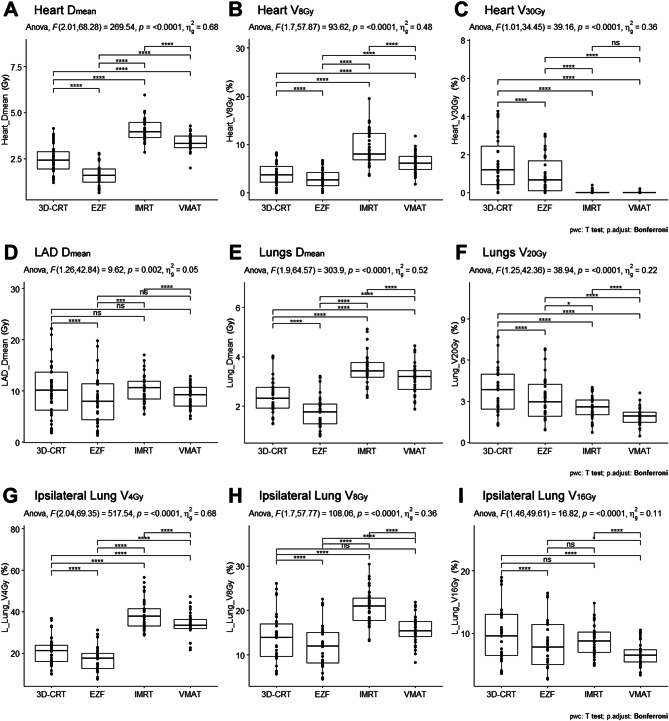
Comparison of the dose exposure of organs. The figure illustrates the comparison of the dose exposure of organs at risk using different radiotherapy techniques. 3D-CRT, three-dimensional conformal radiotherapy; EZF, EZFluence; IMRT, intensity-modulated radiation therapy; VMAT, volumetric-modulated arc therapy. Significance was considered as follows: *adjusted *p* < 0.05; **adjusted *p* < 0.01; ***adjusted *p* < 0.001; and ****adjusted *p* < 0.0001; ns, not significant

The repeated-measures analysis of variance of the four planning techniques revealed significant differences in parameters related to cardiac exposure (Table 3; Fig. 3A–C). High-dose cardiac exposure (heart V_30Gy_) obtained using EZF was higher than that obtained with VMAT and IMRT (adjusted *p* < 0.0001), although it was significantly lower than that when using 3D-CRT (adjusted *p* < 0.0001). An evaluation of the heart D_mean_ indicated that EZF resulted in the lowest overall cardiac exposure (F = 269.54; *p* < 0.0001); in addition, we consistently obtained the lowest values for heart D_5_ and heart V_8Gy_ using EZF compared with those obtained using IMRT, VMAT, and 3D-CRT (adjusted *p* < 0.0001). In contrast, the use of IMRT was associated with the highest heart D_mean_ and heart V_8Gy_ values than were VMAT, EZF, and 3D-CRT (adjusted *p* < 0.0001).

Compared with the other three techniques, significantly lower values of LAD D_max_ were obtained using VMAT; notably, the values obtained with IMRT, EZF, and 3D-CRT were not significantly different (Table 3). In contrast, with respect to LAD D_mean_, significantly lower values were obtained using EZF compared with those obtained using 3D-CRT and IMRT, whereas we detected no significant difference between the values obtained with EZF and VMAT (Table 3; Fig. 3D). Compared with 3D-CRT and IMRT, the use of EZF led to the lowest lung D_mean_ values, contributing to a significant reduction in overall pulmonary exposure (adjusted *p* < 0.0001) (Table 3; Fig. 3E), whereas in the case of the lung V_20Gy_, lowest values were obtained using VMAT, followed by EZF, IMRT, and 3D-CRT (adjusted *p* < 0.0001) (Table 3; Fig. 3F).

In the case of the ipsilateral lung, we observed significant differences among the four assessed treatment techniques with respect to the values obtained for V_4Gy_, V_8Gy_, and V_16Gy_ (Table 3; Fig. 3G–I). Using EZF, we obtained values of 17.12% ± 5.96% and 11.87% ± 4.89% for V_4Gy_ and V_8Gy_, respectively, which were significantly lower than those obtained using the other three techniques (adjusted *p* < 0.0001), whereas the values of these two parameters obtained using IMRT and VMAT were significantly higher than those obtained with 3D-CRT and EZF (Fig. 3G, F). The minimum V_16Gy_ value was obtained using VMAT (6.61% ± 1.84%); although not significantly lower than that obtained using EZF (adjusted *p* < 0.05).

### Contralateral exposure

**Fig. 4:**
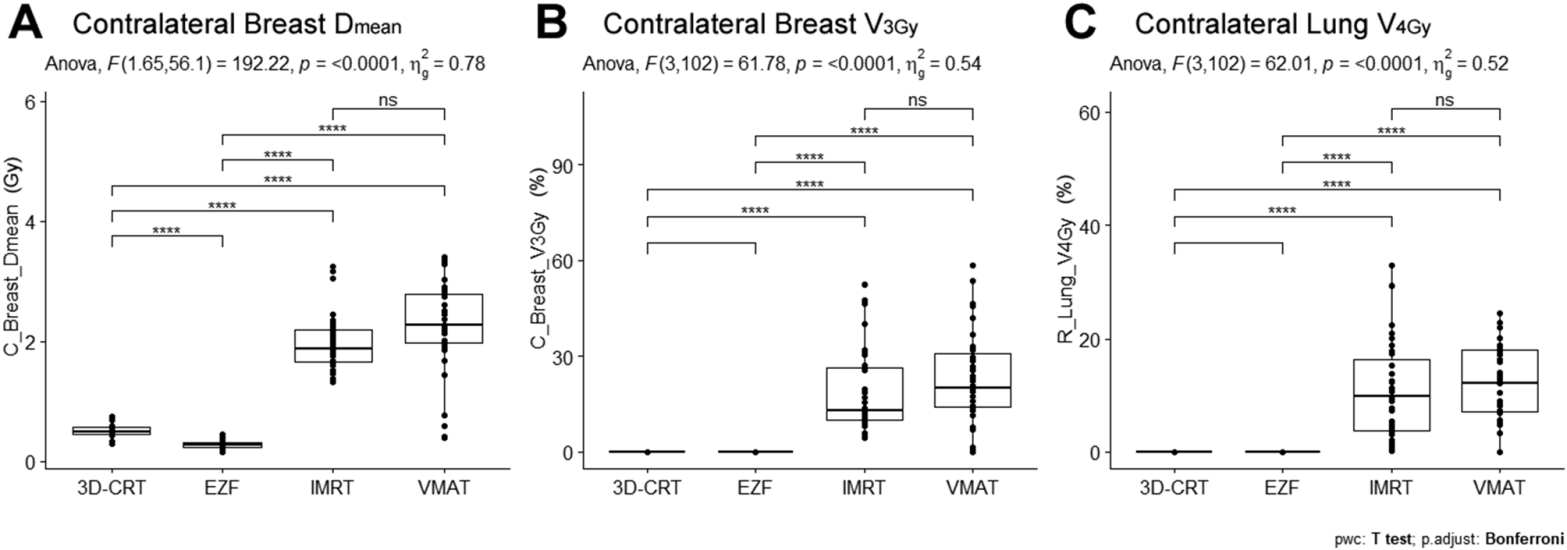
Comparison of the dose exposure of the contralateral breast and right lung. The figure depicts the comparison of the dose exposure of the contralateral breast and right lung using different radiotherapy techniques. 3D-CRT, three-dimensional conformal radiotherapy; EZF, EZFluence; IMRT, intensity-modulated radiation therapy; VMAT, volumetric-modulated arc therapy. Significance was considered as follows: *adjusted *p* < 0.05; **adjusted *p* < 0.01; ***adjusted *p* < 0.001; and ****adjusted *p* < 0.0001; ns, not significant

When using 3D-CRT, EZF, IMRT, and VMAT, we obtained values of 0.53 ± 0.11, 0.28 ± 0.07, 1.96 ± 0.48, and 2.25 ± 0.78 Gy for the mean doses to the contralateral breast (Contralateral Breast D_mean_), respectively (Table 3; Fig. 4A). The values obtained using IMRT and VMAT were significantly higher (adjusted *p* < 0.0001) compared with those obtained using 3D-CRT and EZF. Notably whereas when using 3D-CRT and EZF, we obtained values of zero for the contralateral breast V_3Gy_, significantly higher values were obtained when using IMRT and VMAT (Table 3; Fig. 4B). Moreover, we detected incremental increases in the values obtained for the contralateral breast D_max_ in the order EZF < 3D-CRT < VMAT < IMRT, when using these modalities respectively, with statistically significant differences among the different techniques (adjusted *p* < 0.0001) (Table 3). In addition, IMRT and VMAT were found to be associated with significantly higher contralateral lung doses (Contralateral Lung V_4Gy_), whereas the values obtained using EZF and 3D-CRT were negligible (adjusted *p* < 0.0001) (Table 3; Fig. 4C).

### Planning and delivery efficiency

**Fig. 5:**
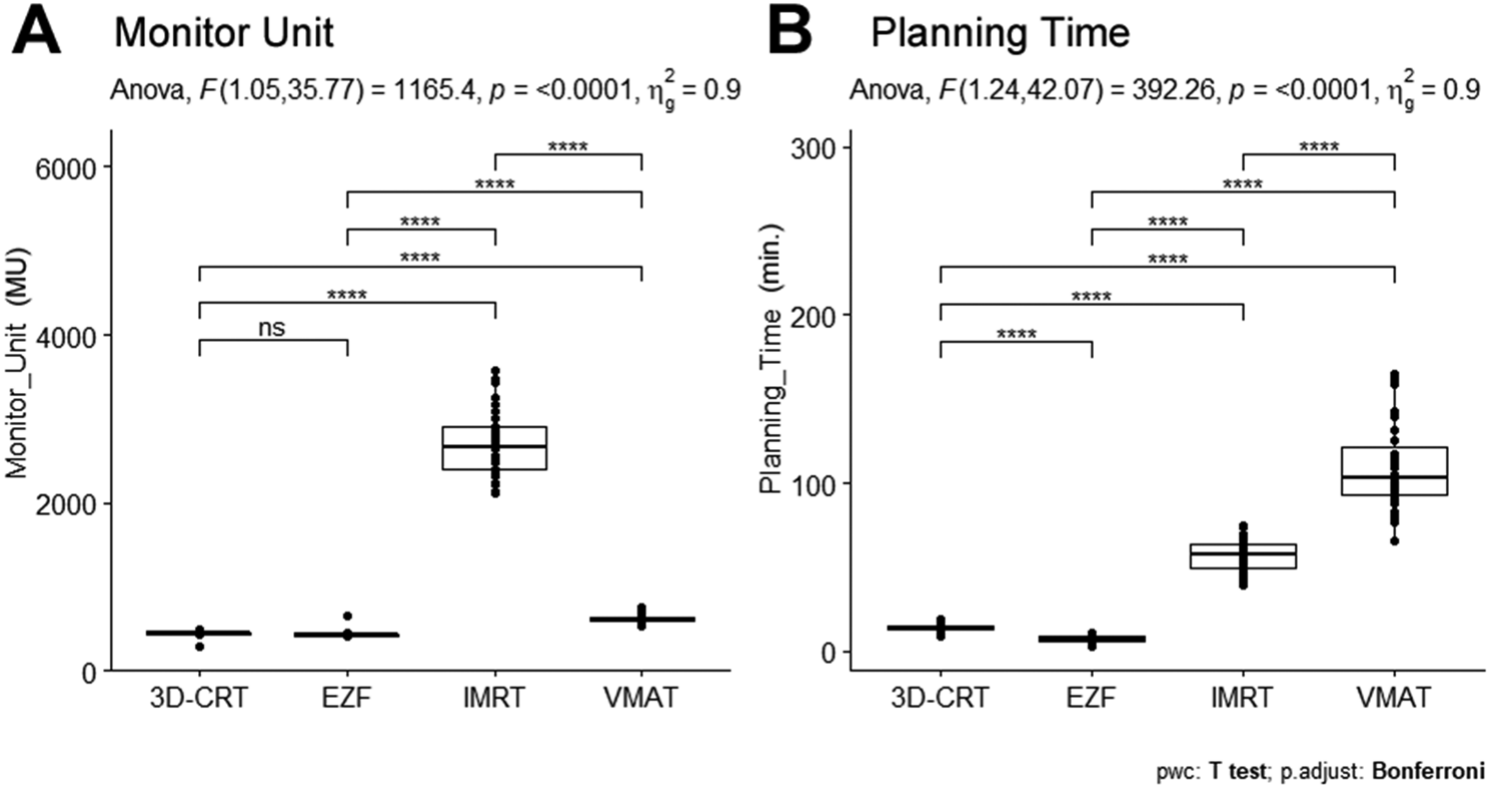
Comparison of monitor units and planning times. The figure shows the comparison of monitor units and planning times using different radiotherapy techniques. 3D-CRT, three-dimensional conformal radiotherapy; EZF, EZFluence; IMRT, intensity-modulated radiation therapy; VMAT, volumetric-modulated arc therapy. Significance was considered as follows: *adjusted *p* < 0.05; **adjusted *p* < 0.01; ***adjusted *p* < 0.001; and ****adjusted *p* < 0.0001; ns, not significant

The results for planning efficiency (planning time) and delivery efficiency (Monitor Units) are summarized in Table 3; Fig. 5.

The MUs of 3D-CRT and EZF were significantly lower than those of IMRT and VMAT (Table 3; Fig. 5A), with no significant difference being observed between the MUs of 3D-CRT and EZF. The highest MU value (2691.89 ± 387.69) was obtained when using IMRT, which was four to six times higher than the values obtained using the other techniques (adjusted *p* < 0.0001).

In addition, we established that the four assessed techniques differed significantly with respect to the total planning time (F = 392.26; *p* < 0.0001) (Fig. 5B). Specifically, on average, the EZF-based plans were completed within 7.24 ± 2.06 min, whereas plans based on 3D-CRT, IMRT, and VMAT were completed within 13.98 ± 2.37, 56.98 ± 9.71, and 109.72 ± 25.95 min, respectively (adjusted *p* < 0.0001). Notably, these findings correspond to seven- to nine-fold reduction in planning time compared with the planning times associated with more modulated techniques.

## Discussion

The ongoing advances in breast cancer radiotherapy have been driven to a large extent by the continuous evolution of planning techniques that aim to optimize target coverage and minimize exposure of the surrounding normal tissues to radiation. Initially, 3D-CRT was widely used as a standard planning approach, comprising wedged tangential fields, to achieve a relatively uniform dose across the breast. However, the limitations of 3D-CRT, including hot spots within the PTV and an increase in high-dose regions in OARs, led to the development and adoption of more advanced techniques, such as IMRT and VMAT, which enable sophisticated dose modulation, thereby enhancing dose conformity and reducing the exposure of OARs to excessively high doses of radiation. However, despite these improvements, application of the IMRT and VMAT techniques necessitates complex inverse planning processes and involves prolonged treatment time, thereby resulting in higher MU usage, which may adversely influence the efficiency of clinical workflows [22–24].

To address these challenges, EZF has been introduced as an automated forward-planning field-in-field technique, designed to balance planning efficiency with high dosimetric quality. In this study, to assess the practicality of applying EZF for breast cancer radiotherapy, we compared the performance of this technique with those of 3D-CRT, IMRT, and VMAT. The results indicated that EZF can be used to provide excellent dose homogeneity; furthermore, EZF achieved significantly lower PTV V_105_ and PTV D_max_ values compared with those achieved with IMRT and 3D-CRT. Although among these techniques VMAT provides the most comprehensive target coverage (PTV V_95_), EZF maintains an optimal balance between target conformity and practical implementation, thus establishing this planning technique as a viable alternative to IMRT and VMAT.

Beyond target coverage, a further key aspect of treatment planning is the sparing of OARs to reduce the likelihood of radiation-induced toxicity. Among the assessed techniques, EZF significantly reduced the dose exposure of the heart and yielded the lowest heart D_mean_. Given the well-documented relationship between the radiation dose to heart and a heightened cardiovascular risk, the reduction in the heart dose obtained using EZF is clinically relevant, particularly for patients with left-sided breast cancer, who are at a greater risk of late cardiac toxicity [2, 25, 26]. However, an evaluation of high-dose exposure (heart V_30Gy_) indicated that VMAT provides superior cardiac sparing, whereas EZF showed significantly higher values. Nevertheless, it is crucial to note that the heart V_30Gy_ levels in EZF plans remained well within clinically accepted safety thresholds (QUANTEC) [27]. Therefore, while VMAT excels in high-dose conformity, the elevated V_30Gy_ in EZF is considered a justifiable trade-off, especially given its superior performance in the low-dose region (V_8Gy_). These findings indicate that although VMAT remains the most effective option for high-dose cardiac sparing, EZF can still be applied to facilitate meaningful dose reductions compared with those obtained using either IMRT or 3D-CRT. Notably, however, EZF demonstrated the lowest low-dose cardiac exposure levels (heart D_5_ and heart V_8Gy_), thereby outperforming all the other assessed techniques. Comparatively, VMAT was found to be characterized by the highest low-dose spread attributable to its continuous arc delivery, thus confirming a key trade-off between high-dose sparing and low-dose dispersion [28, 29]. These dosimetric differences are rooted in the distinct modeling approaches of each technique. EZF employs an automated forward-planning algorithm that specifically generates sub-fields to compensate for missing tissue and thickness variations, inherently prioritizing dose homogeneity and reducing hot spots. In contrast, inverse planning algorithms (IMRT/VMAT) must solve complex mathematical problems to balance competing constraints between target coverage and OAR sparing. This process often results in the optimizer introducing heterogeneous dose distributions or spreading low-dose radiation (integral dose) across a larger volume to achieve the specified high-dose constraints.

In terms of pulmonary dose metrics, IMRT and VMAT resulted in the highest exposure of the lungs to low-dose radiation (ipsilateral lung V_4Gy_ and ipsilateral lung V_8Gy_), consistent with the findings of previous studies that have reported an elevated lung dose using IMRT, attributable to beam modulation and multiple fields [30]. However, with respect to higher-dose lung exposure (ipsilateral lung V_16Gy_), the values obtained using EZF were found to be more comparable to those associated with 3D-CRT, indicating that in certain cases, this technique might not offer the same degree of lung sparing as that offered by VMAT. Nevertheless, we established that EZF maintains a favorable balance between limiting both low-dose and high-dose lung exposure, thus making it an appropriate option for patients at a higher risk of pulmonary complications.

Furthermore, unlike previous studies [16, 17, 19], in this study, we performed a detailed analysis of LAD dosimetric parameters, thereby offering new insights into how different radiotherapy techniques influence exposure of the LAD to radiation. These findings indicated that whereas the lowest LAD D_max_ values were obtained using VMAT, they also indicated that the LAD D_mean_ values obtained using EZF were significantly lower than those obtained using the other assessed techniques. These findings thus indicate that the LAD dose is influenced by multiple patient-specific anatomical factors, such as heart proximity, breast size, and chest wall shape, rather than solely by treatment technique.

In this study, whereas we obtained mean doses to the contralateral breast values of less than 1 Gy when using 3D-CRT and EZF, values in excess of 1 Gy were obtained with IMRT and VMAT (1.96 ± 0.48 Gy and 2.25 ± 0.78 Gy, respectively). In this regard, Watt et al. have reported that an estimated 17% of radiation-associated contralateral breast cancer cases could be prevented by reducing the dose to the contralateral breast to < 1 Gy among women younger than 40 years of age [31]. Consequently, although IMRT and VMAT provide excellent target coverage and OAR sparing, the increased risk of secondary malignancies attributable to unnecessary contralateral breast exposure (mean dose > 1 Gy) should be considered. The findings of previous studies that have compared 3D-CRT with IMRT and VMAT have indicated that 3D-CRT typically results in a lower contralateral breast dose, which can be ascribed to the use of fewer beam angles [32, 33]. However, this benefit tends to be offset by the poorer dose homogeneity and reduced target conformity associated with this technique. Notable advantages of EZF are its capacity to provide a substantially lower contralateral breast dose (0.28 ± 0.07 Gy) and maintain favorable target coverage and OAR sparing. Our findings confirm that EZF retains the dosimetric benefits of advanced planning techniques and substantially reduces unintended contralateral breast exposure.

In terms of efficiency, the application of EZF was associated with a notably reduced MU usage compared with that when using either IMRT or VMAT, and it achieved values comparable to those obtained using 3D-CRT. A lower MU requirement confers multiple benefits, including shorter treatment times, lower dose scatter, and a reduced overall burden on treatment equipment. A significant challenge associated with highly modulated techniques such as IMRT and VMAT is a heightened sensitivity to patient movement and respiratory motion, which can introduce dose delivery uncertainties [34–36]. Breast radiotherapy, in particular, is susceptible to respiratory-induced motion, and studies in this regard have indicated that compared with highly modulated techniques, simpler field-in-field-based techniques such as EZF are less sensitive to motion-related dose variations [37]. Thus, these findings indicate that EZF could serve as a more robust and reproducible treatment option, particularly for patients who are unsuitable for deep inspiration breath-hold or other motion-management strategies.

Moreover, we found that EZF contributed to a significant shortening of the total planning time, compared with IMRT and VMAT, thereby highlighting its capacity to enhance workflow efficiency by alleviating the workload of planners and maintaining high dosimetric quality. Such advantages are of particular importance in high-volume clinical settings, in which rapid and reliable plan generation is essential for sustaining patient throughput.

It is important to note that the clinical impact of this time reduction extends beyond merely accelerating the start of treatment for a single patient. While logistical factors such as machine availability often dictate the treatment start date, the significant reduction in planning time from approximately one hour to under ten minutes creates substantial operational elasticity. This efficiency releases dosimetric resources, allowing planners to allocate more time and focus to other complex cases that necessitate intensive manual optimization. Furthermore, the rapidity of EZF significantly lowers the practical barrier to plan modifications, allowing for prompt plan revision without disrupting the clinical workflow should anatomical variations arise.

Admittedly, the landscape of radiotherapy planning is rapidly evolving, with recent advancements in GPU-based optimization significantly reducing calculation times for inverse planning techniques like IMRT and VMAT. However, while high-performance computing minimizes the computational burden, it does not fully eliminate the time required for manual objective setting and iterative plan refinement. In contrast, EZF streamlines the entire workflow by automating fluence optimization with minimal user intervention, effectively eliminating the need for iterative manual parameter adjustments inherent in inverse planning. Thus, even in the era of high-speed computing, the reduction in human workload and inter-planner variability offered by EZF remains a distinct and valuable advantage.

In the context of maximizing clinical resources, the choice of the treatment delivery system is also a relevant factor. While the Varian Clinac iX used in this study represents an established generation of linear accelerators compared to the most recent platforms, it continues to serve as a primary treatment unit in numerous institutions worldwide. Consequently, optimizing workflows for such widely deployed systems remains a critical clinical priority. Our findings demonstrate that integrating automated fluence optimization software like EZF can significantly enhance the capabilities of this standard platform. By achieving dosimetric outcomes comparable to VMAT and reducing planning time, EZF offers a practical strategy to elevate treatment quality on existing infrastructure without necessitating immediate hardware upgrades. Future studies are warranted to evaluate the performance of EZF on next-generation delivery platforms.

Given its promising dosimetric and clinical advantages, future research should focus on expanding the application of EZF beyond standard whole-breast radiotherapy. Potential areas of interest include hypofractionated and ultra-hypofractionated breast radiotherapy, which are currently gaining widespread adoption on account of their capacity to deliver effective treatment in fewer sessions [38–41]. Evaluations of the performance of EZF in these settings will become essential as shorter treatment regimens become more common. Moreover, it is widely anticipated that the integration of artificial intelligence-based treatment planning tools will further enhance the efficiency of EZF [17, 19, 42]. Accordingly, future studies should assess the feasibility of combining artificial intelligence-driven auto-contouring with EZF to automate the entire planning process, thereby further reducing inter-planner variability and treatment planning time. Finally, given the challenges associated with respiratory motion, further investigations of the application of EZF in gated or adaptive radiotherapy could provide valuable insights into its role in motion-compensated treatment delivery.

In summary, our findings highlight the potential utility of EZF as an effective and efficient option in planning breast cancer radiotherapy; moreover, EZF can achieve comparable target coverage, provide enhanced OAR sparing, and minimize unnecessary contralateral exposure compared with more established techniques. Moreover, its capacity to streamline the planning process without compromising dosimetric quality establishes EZF as a valuable addition to contemporary radiotherapy workflows. By reducing treatment complexity and maintaining robust dosimetric performance, EZF addresses key clinical demands in high‑volume settings. With the ongoing advancements in radiotherapy, further investigations of artificial intelligence‑driven automation, hypofractionated and ultra‑hypofractionated regimens, and motion‑adaptive strategies will be essential for expanding the clinical utility of EZF and optimizing the outcomes of patients with breast cancer.

## Conclusions

EZF can be considered a clinically viable alternative to VMAT, IMRT, and 3D-CRT for whole-breast irradiation that can effectively balance target coverage, OAR sparing, and planning efficiency. Application of EZF can significantly reduce the ipsilateral OAR dose and unnecessary contralateral exposure, streamline treatment workflows, and maintain a dosimetric performance comparable to that obtained using VMAT. Given that it simplifies both planning and delivery, EZF represents a practical solution that should be integrated in routine clinical practice, particularly at busy centers that aim to optimize radiotherapy delivery without compromising treatment quality.

## Supplementary Information

Below is the link to the electronic supplementary material.

Supplementary Material 1: Supplementary Figure S1: Comparison of average dose-volume histograms (DVHs) for target volumes and organs at risk. The average DVHs calculated across all 35 patients are presented for (A) PTV, (B) CTV, (C) contralateral breast, (D) lungs, (E) ipsilateral lung, (F) contralateral lung, (G) heart, and (H) LAD. The curves represent the four treatment techniques: 3D-CRT (blue squares), EZF (yellow circles), IMRT (gray triangles), and VMAT (red inverted triangles). 3D-CRT, three-dimensional conformal radiotherapy; EZF, EZFluence; IMRT, intensity-modulated radiation therapy; VMAT, volumetric-modulated arc therapy; PTV, planning target volume; CTV, clinical target volume; LAD: left anterior descending artery.

## Data Availability

The original contributions presented in the study are included in the article. Further inquiries can be directed to the corresponding author.

## Abbreviations

3D-CRT: Three-dimensional conformal radiotherapy
IMRT: intensity-modulated radiation therapy
VMAT: volumetric-modulated arc therapy
OARs: organs at risk
EZF: EZFluence
PTV: planning target volume
LAD: left anterior descending artery
D_mean_: mean dose
D_max_: maximum dose
